# New York Odontological Society

**Published:** 1887-02

**Authors:** 


### NEW YORK ODONTOLOGICAL SOCIETY.

Reported for the Independent Practitioner.

The January meeting of this Society was held at the rooms of the Academy of Medicine on the evening of the 11th ult., Vice President J. M. Howe presiding.

Dr. C. A. Woodward introduced a patient with two inferior central incisors implanted by Dr. Younger last summer. The natural teeth had been out for some years, and the process had become absorbed to such an extent that the bone was only a line or two in thickness, making it seem as if striving for the impossible to implant a tooth with such frail surroundings. The teeth implanted were not satisfactory to Dr. Younger, as the pericementum covered but a small portion of their roots. In a few weeks they came out. Nothing daunted, however, he implanted two others, which are today firm, giving much comfort and satisfaction to the patient, and the gums are in a healthy condition.

Dr. N. W. Kingsley described two interesting cases in which he had the honor to perform the operations, one of implantation and one of replantation. The first, for a lady of uncertain age, had a space between two teeth (bicuspids) which had been gradually widening for some time, but not enough to give room for a natural tooth. Wedges were used until sufficient space was obtained, when a tooth was implanted which had been extracted and imbedded in a plaster model seven or eight years previously, there remaining until the opportunity came to use it for some better purpose. This tooth was implanted several weeks ago, and the operation has proved satisfactory to both patient and operator.

The other case was that of a lady of about the same age as the first, whose superior left central and lateral incisors stood so far out of the arch as to greatly disfigure her, and she earnestly wished their regulation. The doctor informed her that the only possible way to correct the deformity was to extract the teeth and replant them. He therefore extracted them and formed anew the sockets for their reception. Finding the teeth too wide for the space when set back, he ground them narrower until they were well fitted, then forced them to their places. This case had also proved a success. In both cases they were held in position by splints.

Dr. S. G. Perry exhibited a set of band matrices of spring steel (from Ash & Son of London). The ends of the band or loop were brought together and secured by means of a screw, and tightened with a small flexible wrench. The bands and wrench were neatly constructed, and seem well fitted for the purposes intended.

The subject for discussion was here announced by the chair—the paper recently read by Dr. Bonwill, of Philadelphia, on the Herbst method of filling teeth.

Dr. B. Lord gave much credit to Dr. Herbst, whom he highly esteems as a useful member of the profession, yet he does not think very favorably of his peculiar method of filling teeth. One objection cited is, that it requires direct pressure upon every part of the cavity to be filled, and to get access to some of these, especially in proximal cavities, must cause a sacrifice of too much tooth substance. He does not think this method will ever become very popular or be generally adopted. Individually, he prefers the old methods of filling, using soft foil for packing against the walls, and for forming the bulk of the filling, and employing cohesive foil only for finishing the surface or restoring contours. He does not consider the Herbst matrix of much account. He sometimes uses a narrow matrix, but never a wide one, as it obstructs the view of the cavity.

As regards the rubber dam and engine, he thinks they are used too much and too often; many cavities can be prepared and filled equally well without the aid of either engine or dam, and much annoyance may thus be saved to the patient.

Dr. W. H. Dwinelle declared that Dr. Lord in his remarks had about expressed his own sentiments. He does not believe the Herbst method will ever be generally adopted. In some cases he has tried it for partly filling cavities, and with a good degree of success. He was reminded of the statement that "old fogies would not be apt to change their old and tried methods," which probably is the case. The manner in which Dr. Herbst had been received by the dentists of this country should not be construed as adopting his method of practice. He was honored as a man—a decidedly practical man—and it was right that he should have been so warmly welcomed and gracefully treated.

Although many operators at different times had worked gold foil by the "rotary" process to some extent, he gave credit to Herbst for adopting it as a special method, and as based on a theory.

Dr. Dwinelle does not believe that good fillings depend on any particular kind of gold, or foil from any special manufacturer; neither upon the peculiar instruments employed in packing the metal, but rather upon the man or the genius who guides the instrument as it does its good work.

Regarding the idea of testing the solidity of old gold stoppings by passing them through the rolling-mill, the doctor remarked that it was not a true test, for even indifferent fillings, if well cleansed and annealed, will become compact if passed between the rollers.

Dr. W. H. Atkinson said that those who did not understand and had not tried the Herbst method could not intelligently condemn it.

He was an advocate for the heavy mallet, and uses it, yet he saw advantages in the method of Herbst.

